# Preventing childhood obesity during infancy in UK primary care: a mixed-methods study of HCPs' knowledge, beliefs and practice

**DOI:** 10.1186/1471-2296-12-54

**Published:** 2011-06-23

**Authors:** Sarah A Redsell, Philippa J Atkinson, Dilip Nathan, Aloysius N Siriwardena, Judy A Swift, Cris Glazebrook

**Affiliations:** 1 School of Nursing, Midwifery and Physiotherapy, The University of Nottingham, Queen's Medical Centre, B Floor, South Block, Nottingham, NG7 2HA, UK; 2 Nottingham City Care Partnership, 1 Standard Court, Nottingham. NG1 6GN, UK; 3 Nottingham University Hospitals NHS Trust, Nottingham, NG7 2UH, UK; 4 School of Health and Social Care, University of Lincoln, Lincoln, LN6 7TS, UK; 5 School of Bioscience, The University of Nottingham, Sutton Bonington Campus, Loughborough, LE12 5RD, UK; 6 School of Community Health Sciences, The University of Nottingham, Nottingham, NG7 2HA, UK

## Abstract

**Background:**

There is a strong rationale for intervening in early childhood to prevent obesity. Over a quarter of infants gain weight more rapidly than desirable during the first six months of life putting them at greater risk of obesity in childhood. However, little is known about UK healthcare professionals' (HCPs) approach to primary prevention. This study explored obesity-related knowledge of UK HCPs and the beliefs and current practice of general practitioners (GPs) and practice nurses in relation to identifying infants at risk of developing childhood obesity.

**Method:**

Survey of UK HCPs (GPs, practice nurses, health visitors, nursery, community and children's nurses). HCPs (n = 116) rated their confidence in providing infant feeding advice and completed the Obesity Risk Knowledge Scale (ORK-10).

Semi-structured interviews with a sub-set of 12 GPs and 6 practice nurses were audio recorded, taped and transcribed verbatim. Thematic analysis was applied using an interpretative, inductive approach.

**Results:**

GPs were less confident about giving advice about infant feeding than health visitors (p = 0.001) and nursery nurses (p = 0.009) but more knowledgeable about the health risks of obesity (p < 0.001) than nurses (p = 0.009). HCPs who were consulted more often about feeding were less knowledgeable about the risks associated with obesity (r = -0.34, n = 114, p < 0.001). There was no relationship between HCPs' ratings of confidence in their advice and their knowledge of the obesity risk.

Six main themes emerged from the interviews: 1) Attribution of childhood obesity to family environment, 2) Infant feeding advice as the health visitor's role, 3) Professional reliance on anecdotal or experiential knowledge about infant feeding, 4) Difficulties with recognition of, or lack of concern for, infants "at risk" of becoming obese, 5) Prioritising relationship with parent over best practice in infant feeding and 6) Lack of shared understanding for dealing with early years' obesity.

**Conclusions:**

Intervention is needed to improve health visitors and nursery nurses' knowledge of obesity risk and GPs and practice nurses' capacity to identify and manage infants' at risk of developing childhood obesity. GPs value strategies that maintain relationships with vulnerable families and interventions to improve their advice-giving around infant feeding need to take account of this. Further research is needed to determine optimal ways of intervening with infants at risk of obesity in primary care.

## Background

Globally, childhood obesity is a significant public health issue [1]. It is associated with a range of life-threatening and debilitating physical health and psychosocial consequences [2,3] and it is likely to increase the risk of adult obesity [3]. In the UK the prevalence of childhood obesity has increased significantly since 1995 [4], though emerging research shows that the prevalence is levelling off suggesting that rates may have peaked [5]. The most recent rates for childhood overweight and obesity among 2-15 year olds are 31% for boys and 30% for girls [4].

Early childhood is recognised as a key developmental period in which to address the obesity epidemic [6,7] and UK health policy endorses this [8-10]. There is convincing evidence that most excess weight before puberty is gained before 5 years of age [11]. Estimates vary but it is thought that 25% [12]-33% [13] of infants gain weight more rapidly than desirable during the first six months of life. Three systematic reviews have concluded that rapid weight gain during infancy is associated with obesity in later life [14-16]. In addition, two well conducted prospective longitudinal cohort studies show that rapid weight gain, between 0-3 months in a Hong Kong Chinese population [13] and between 0-4 months in a US population [17] is associated with a greater risk of obesity at age 7 years. However, most childhood obesity prevention efforts focus on periods of the life span that follow these early periods, after children's initial growth trajectories and eating habits have already been established [6]. The risk factors for childhood obesity are identifiable [18] and an infant's weight trajectory may be modified by intervention directed at early feeding practices [6].

### Barriers to primary care management of childhood obesity

There is good evidence that healthcare professionals (HCPs) find it difficult to manage children who have become overweight or obese [19-21] and primary prevention during infancy may present an even greater challenge. A systematic review exploring primary care physicians' knowledge, attitudes, beliefs and practices regarding the management of childhood obesity identified three key themes [22]. These were related to knowledge deficits, in particular low reported use of guidelines, low levels of self-perceived competency to treat childhood obesity, inconsistent use of standard measures such as BMI and lack of clinical consensus around treatment [22]. In the UK a recent qualitative study reported that although general practitioners (GPs) and practice nurses consider it their role to raise the issue of a child's weight they felt unable to cope with the scale of the problem and doubted the effectiveness of giving advice about diet and exercise [20]. They also report concerns about the sensitive nature of the subject and the negative effect that bringing attention to a child's weight might have on their relationship with the parent [20].

### Primary obesity prevention

There have been few studies examining obesity prevention during infancy or early childhood in primary care. A qualitative study explored the views of HCPs working with a primary target population of low income mothers in the US [23]. HCPs perceive that mothers focus on surviving daily life stresses and use child-feeding to help cope with parenting. The authors recommend that HCPs working with this population receive more focusing training in counselling rather than nutrition skills [24]. A US study explored parents' views of a primary-prevention interventions in 5-10 year olds [25]. Activities such as BMI screening and targeted behaviour counselling were not perceived as normal practice for HCPs, although parents reported they found the take-home messages about diet and weight motivating for behaviour change [25].

In the UK NICE guidance recommends cooperation between NHS managers and HCPs (including GPs), local authorities and early years settings (nurseries and care centres for 0-5 year olds) to prevent childhood obesity [9]. It states that "families of children and young people identified as being at high risk of obesity - children with at least one obese parent - should be offered ongoing support from appropriately trained healthcare professionals" (page 21) [9]. Unfortunately the content and timing of this support are poorly defined, reflecting limited clinical consensus and an absence of proven early years preventative interventions [6]. Furthermore the knowledge of UK primary HCPs in relation to the health risks of obesity has not been previously reported. If their obesity risk knowledge is low, HCPs may not deliver appropriate feeding advice to parents of infants at risk of obesity. Given the evidence supporting a need for early intervention [6], this study, aimed to explore obesity-related knowledge of UK HCPs and the beliefs and current practice of GPs and practice nurses in relation to identifying infants at risk of developing childhood obesity. There were two main research questions:

1. How does knowledge of the health risks associated with obesity compare between HCPs working in primary care (GPs, practice nurses, health visitors, nursery, community and children's nurses)?

2. What are the beliefs and current practices of GPs and practice nurses in relation to the identification and management of infants at risk of developing childhood obesity?

## Method

### Sampling and recruitment

HCPs working in two counties in the East Midlands region of the UK were sampled during 2008/2009. Five sites were selected with high rates of childhood obesity and one with low levels of obesity in the practice population; a detailed outline of the demographics of each site can be found in Redsell and colleagues (2010) [26]. Different study sites were used to ensure a range of views from HCPs working in rural/urban, deprived/affluent areas were included in the qualitative aspect of this study. The sampling strategy was purposive to include HCPs from all groups involved in service delivery for infants (GPs, practice nurses, health visitors, nursery, community and children's nurses). Study registration with the Primary Care Research Network (PCRN) helped identify and recruit general practices (GPs and practice nurses). Other HCPs (health visitors, nursery, community and children's nurses) were identified through PCT locality managers. Service support costs were available to all study participants.

There were two study phases: a survey and semi-structured telephone interviews. All HCPs working in the study sites were eligible to participate in both phases and were invited by personalised letter, accompanied by an information sheet, survey, a reply slip to express interest in telephone interviews and a freepost envelope (n = 243). The PCRN and the health visiting managers also circulated emails with an on-line version of the survey.

Nottingham 1 Research Ethics Committee 08/H0403/3 and Nottinghamshire and Lincolnshire Primary Care Trusts (PCTs) approved the study. All HCPs interviewed for this study provided their written, informed consent. At the time of data collection, the local Research Ethics Committees did not require consent specifically for sharing the raw data from the interview manuscripts. The presented quotes are anonymised and risk of identification is low.

### Data collection and analysis

#### Phase 1 Survey

The survey requested HCPs practice postcode, personal details including age, gender and ethnicity and professional qualifications, training and number of years in practice. Participants rated their confidence in relation to infant feeding advice and completed the Obesity Risk Knowledge Scale (ORK-10): a reliable, discriminating and valid 10-item scale assessing knowledge regarding the effects of obesity on health [27]. The ORK-10 scale is a self-report questionnaire requiring 'True' or 'False' answers and has been successfully used to assess the knowledge of practising and trainee health care professionals, community and clinic samples [28,29]. Data distortion was reduced by allowing a 'don't know' option for areas of uncertainty. Responses were treated as dichotomous variables where correct responses score one point, while incorrect and don't know responses score zero. Scores on the ORK-10 scale range between zero and 10 with higher scores indicating higher levels of knowledge. Data were analysed using non-parametric statistical tests facilitated by SPSS software (version 15.0) [30].

#### Phase 2 Interviews

The purpose of the interviews was to ascertain the beliefs and current practices of GPs and practice nurses in relation to identifying and managing infants at risk of childhood obesity. The interview guide was informed by previous literature [31] (Box 1) and was piloted with two GPs not involved in the study. They advised the research team to remove specific questions around infant feeding and weaning which they felt were not relevant for practice staff and might alienate participants. These items were included in the interview guide used with members of the health visiting team, the findings of which are not reported here. The telephone interviews were semi-structured and were conducted by a health visitor (PA) and a community paediatrician (DN).

Telephone interviews lasting up to an hour were audio-recorded and transcribed verbatim by a member of the research team. Interview data were analysed thematically using an interpretative perspective in which the researchers looked beyond the spoken word in order to the understand meaning behind the dialogue [32]. The transcribed interviews were entered into NVIVO 8.0 [33]. Two researchers independently coded five of the transcripts using an inductive approach [32] to ascertain preliminary codes. The codes were arranged into related clusters to form a coding frame which was then explored for linked and explanatory themes [34]. A coding book was developed following the guidelines set out by Boyatzis (1998) [35] which comprised codes, definitions and examples. An inter-rater reliability check conducted on 10% of the coded data revealed 100% agreement on theme identification, however, discussion between the research team resulted in some further adjustment of the coding frame. Data were re-analysed as appropriate within the revised framework.

## Results

### Phase 1 Survey

One hundred and eighteen postal survey questionnaires were returned within the study period representing GPs, practice nurses, health visitors, nursery, community and children's nurses. Eighty-four participants responded to the initial postal survey (n = 243), a response rate of 34%, and a further 34 were recruited via the on-line survey. We do not have a denominator for the number of individuals who may have received an invitation to complete the on-line version of the survey via email.

As there were only a few responses from children's and community nurses, their responses were combined with practice nurses to form a single 'registered nurse' group. Two respondents were excluded as they could not be allocated to a professional group, giving a useable sample of 116. Most participants were experienced HCPs having been in practice for at least 10 years (Table 1).

**Table 1:** Prompt guide for semi-structured interviews

**Questions about healthcare professionals' current practice, training needs**
Can you describe your role in relation to discussing diet with parents of children under 1 year old?
How do you think parents of children under 1 years old feel about your dietary advice?
Are there any aspects of your advice that parents are reluctant to implement or find difficult to implement?
Are there any parent characteristics that make it more likely that they will follow your advice about diet in children under 1?
Have you undertaken any training in relation to providing advice about diet to children under 1 year old?
**Questions about identification of 'infants at risk' and healthcare professionals strategies**
What do you think are the most important risk factors for childhood overweight/obesity?
Do you have infants or young children on your caseload that you think might become overweight/obese children?
How do you manage situations where an infant's growth increases disproportionately on the percentile charts?
For example, if an infant's growth went up from the 50^th ^to the 90^th ^percentile or crosses three percentiles with no corresponding rapid growth in head circumference or length?
What else might you need to know?
Have you found anything that makes it difficult to discuss with parents that their infant or young child is 'at risk' of becoming overweight?
What would help you to overcome this?
How do you find dealing with parents whose infants are overweight/obese?
**Questions about weight management and intervention**
Can you describe any intervention you have initiated with parents whose infants or young children have grown too quickly?
How might healthcare professionals work with parents of infants 'at risk' to prevent childhood overweight/obesity?
What type of intervention might be needed?
What information should an intervention contain to make it useful for healthcare professionals to use with parents?
In your view how could the information be presented clearly and consistently by different members of the PHCT?
Is there anything else you would like to mention that you think would be useful?
Thank you

All participants had been consulted by parents for advice about infant feeding. Nursery nurses (Fisher exact probability = 0.009) and health visitors (χ^2^_(1) _26.35, p < 0.001) were significantly more likely to have been consulted at least weekly compared to other professions. GPs (χ^2^_(1) _= 9.5, p = 0.002) and registered nurses were less likely to have been consulted (χ^2^_(1) _= 7.3, p = 0.006). Self-rated confidence in providing infant feeding advice differed significantly between professional groups (Kruskall Wallis χ^2^_(3) _= 19.16, p < 0.001). Health visitors were significantly more confident about the advice they provided than GPs (post-hoc Mann Whitney test Z = 3.44, p = 0.001) and nurses (Z = 3.92, p < 0.001). Knowledge about health risks associated with obesity, as measured by the ORK-10, also differed significantly between groups (χ^2^_(3) _= 26.7, p < 0.001). Health visitors were more knowledgeable about the health risks of obesity than nursery nurses (Z = -2.6, p = 0.009) but less knowledgeable than GPs (Z = 4.26, p < 0.001) who had a median score of 9/10. GPs were also more knowledgeable than registered nurses (Z = -2.59 p = 0.01) and nursery nurses (Z = -4.27, p < 0.001). Registered nurses were more knowledgeable than nursery nurses (Z = -2.85, p = 0.004) (Table 2). Overall, participants who were most frequently consulted around infant feeding were less knowledgeable about the risks associated with obesity (r = 0.34, n = 114, p < 0.001). There was no relationship between HCPs self-rated confidence and their knowledge about obesity risk (Table 3).

**Table 2:** Demographic characteristics of sample

Gender	
Male	19 (17.4%)
Female	97 (83.6%)
**Age**	
**20-29**	**7 (6.0%)**
**30-39**	**21 (18.1%)**
**40-49**	**46 (39.7%)**
**50-59**	**36 (31.0%)**
**60+**	**6 (5.2%)**

Profession	
Nursery nurse	8 (6.9%)
Health Visitor	27 (23.3%)
Nurse (practice nurse or community nurse)	29 (25.0%)
General Practitioner	52 (44.8%)

**Years of Experience**	
**Less than one year**	**1 (0.9%)**
**1-5**	**23 (19.8%)**
**5-10**	**23 (19.8%)**
**10+**	**69 (59.8%)**

**Table 3:** Comparison between professional groups for dependent variables

	Nursery nurses (n = 8)	Health visitors (n = 27)	Nurses (n = 29)	GPs (n = 52)
Consulted about infant feeding at least once a week	8 (100%)	27 (100%)	9 (31.1%) ^a^	20 (39.2%)^a^

Confidence about giving feeding advice				
Very confident	2 (25%)	4 (14.8%)	2 (7.1%) ^a^	4 (7.8%) ^a^
Confident	5 (62%)	23 (85.2%)	11 (39.3%)	27 (52.9%)
Slightly confident	1 (12.5%)	0 (0%)	13 (46.4%)	19 (37.3%)
Not confident	0 (0%)	0 (0%)	2 (7.1%)	1 (2.0%)

Knowledge about risks of obesity Median (IQR)	5.5 (4.25-6.75)	7 (5-8)	8 (6-9)	9 (8-9)

IQR = Inter Quartile Range ^a ^1 missing value

### Phase 2 interviews

Telephone interviews were conducted with 12 GPs (n = 6 male, n = 6 female; n = 7 white British, n = 2, British Asian, n = 1 Middle Eastern, n = 2 British Indian) and 6 practice nurses (all female, white British).

#### 1. Attribution of childhood obesity to family environment

Participants viewed childhood obesity as having a genetic/biological origins but causation was also attributed to economic, educational and emotional poverty within families.

*I think an obese parent is certainly a factor, I think poor socio-economic groups tend to have less knowledge on diet, as well as less money "to", they think that a healthy diet is more expensive. I do feel that a lot of young parents now haven't learnt how to cook, from basics. PN213*.

I think yeah I think poor social circumstances as well and diet because I think from what I've seen I think the children poor, but when I mean poor in social circumstances I'm not talking about money, I'm talking about a family unit. GP211.

Participants did not identify infants who were obese but considered that some were over fed, particularly those on formula milk who were weaned early.

Over feeding... early weaning would count as overfeeding. Over feeding I would think was the most important factor. GP240.

There was a strong belief that parent's feeding strategies were adversely influenced by their peers and family, particularly grandparents.

They seem to have a lot of input from older generations, who say, oh that baby's hungry, it should be weaned, and you know, going back to when they used to put a Farley's Rusk in the bottle I think! So I talk to them about inappropriately weaning the baby because I think what a lot of mum's who find it quite hard work having a young baby, they can get quite a lot of messages from extended family that weaning the baby will settle it, help it sleep through the night and make it more content. GP241.

#### 2. Infant feeding advice as the health visitor's role

There was a strong belief that the role of general practice is to manage non-routine problems or illness and those who expressed that view denied any knowledge about appropriate infant feeding.

My recent experiences have been constipation and reflux. Weaning, I can't remember anything about the weaning thing. GP211.

Participants believed that infant feeding advice was the responsibility of the health visiting team, who have traditionally led the way in advising parents about infant feeding [36].

*Have very little err involvement in under one year olds. I tend to recommend that the parents take the queries to the health visitor erm because I think it is really important that we don't end up with conflicting advice cos that's the worst thing you can do when you've got a new baby. GP203*.

*I don't really as a Practice Nurse deal with under ones as a dietary thing, it would be the Health Visitor. PN217*.

However, there was a minority view that GPs should actively promote healthy infant feeding although this was not supported nor expressed by any of the practice nurse participants.

*One of the most important positive things you can put across to parents with the under ones is that now is the best time to tackle it because if you can tackle it then you know the under ones still eat what they're given, they don't eat what they take, they eat what they're given, you can start to reverse the trend. GP240*.

Obesity prevention and management during early years was seen as the role of the health visitor or other specialist practitioner not the role of the GP or practice nurse.

*Who should deliver it, well the health visitor would either deliver it herself or she would know where parents could go to get the information. GP240*.

#### 3. Professional reliance on anecdotal or experiential knowledge about infant feeding

Participants suggested that obesity services were targeted at adults and identified a lack of training for childhood obesity prevention and management.

*And there isn't any training available, it's not like being sat there, folding our arms, and saying oh dear we don't know what we're doing, there isn't anything that I know of that we can go on, otherwise we'd all have been on it by now I think. We've all been on endless rounds of adult training, and adult intervention for obesity till we're purple. PN219*.

Participants described how the information they used to support the advice they provided to parents about infant feeding was gained anecdotally or experientially.

I guess just general reading, like the GP magazine, some parenting books which I have read a few of, discussion with my wife and other colleagues, other colleagues with young children. But no formal training at all. GP241.

And quite often it's, it's not just being as a GP it's more like a maternal thing in my experience as a mum really that's been able to guide them. GP211.

Participants felt that little guidance and training was available to them. Some, but not all, participants were happy to provide infant feeding advice in response to parent's requests.

Like I mentioned I never had any training, but I'm giving advice, and I'm sure there's lots of other people in the same position. GP251.

*If I was more clued up on what information to give I would feel happy to do that so it would boil down to education I think. PN217*.

#### 4. Difficulties with recognition of, or lack of concern for, infants "at risk" of becoming obese

There was a lack of explicit recognition that infants could be identified as overweight or obese.

*I personally, currently don't have any patients that I thought were, are particularly high at risk, at the present time. GP250*.

However, participants acknowledged that some infants demonstrated excess weight gain but lacked common terminology to describe this. Participants expressed the view that intervening with overweight or obese infants during non-routine or illness consultations was problematic for a number of reasons. GPs suggested it was often difficult to intervene with the parents of these infants because they had no visible growth record available to them. The parent-held records which contain information about infant feeding and growth were not usually available during a non-routine or illness consultation. Therefore their responsibility for following up/reinforcing infant feeding advice provided by others was negated.

Most of them don't bring them [parent held record or red books] unfortunately, but yes, definitely I've seen a few babies that look a bit larger than they should be. GP251.

*They don't come in with their red books, so quite often I don't know what centile they're on so you know you just see a baby and it looks sitting on mum's lap, at one a lot of them are walking and he just looks chubby. GP211*.

There was reluctance to target at risk infants for preventative activities.

To be honest I haven't looked specifically at targeting that age group. GP242.

Participants described how they might mentally note the weight of an infant but would not intervene until they were older and already overweight or obese.

So I think there are some families that you can identify where the children already you can see are slightly carrying more weight than they should do. But I don't think necessarily that you see that as an under one. I don't feel I can see that as an under one child, these are usually around sort of four year olds onwards that you start to really see. GP239.

#### 5. Prioritising relationship with parent over best practice in infant feeding

Participants were aware of the guidance supporting best practice in infant feeding but some of them made exceptions with parents whom they perceived to be struggling to cope. They described how some parents found it difficult to manage their infant's needs and to reduce the demands made on them they adapted the care they provided. This might involve soothing infants with additional food and/or weaning early to ensure the parent has a good night's sleep.

In this practice we have a lot of single mums, young mums, unsupported mums, not particularly good accommodation, and they've got the baby 24/7 with either no support from boyfriend or a boyfriend who's a waste of space, one of the things that wears them down incessantly is sleep disturbance because they don't get the help, they're concerned if the baby's crying all night, the neighbours complain, or the neighbours think they're not looking after the baby, etc., etc., they've got a very high aim to get the baby to sleep well, so they will feed the baby in the hope of it sleeping, and I think they also see giving the baby solids early as a way of getting the baby to sleep. GP240.

GPs were sympathetic to these parents and supported some of their decisions especially in relation to deviating from the Department of Health's current weaning guidance [36]; a position reflected in a related study of parents' views.

*They are only guidelines and you will get babies that have been born at nine pounds that no way can they cope 'til they're six months without some solid food erm it's just you have to be sensible about things and you know erm there are lots of issues people will have had children years ago where you know we weaned much earlier and they'll be in-laws interfering and you need to be aware of every individual's personal situation, can't have a hard and fast rule but err you know certainly I always recommend they try and wait until the baby's six months but I don't ban people from weaning sooner if they have a desire to. GP203*.

There was also a perception that identifying infants as overweight or obese would be stigmatising and might jeopardise the professional relationship with the parent.

*I would, although as I've said I haven't that particular situation, I would just find that embarrassing and difficult to bring up, and I think it would be the fact that they would feel I was criticising them and their parenting, or their overweightness, if that was the risk factor. GP218*.

#### 6. Lack of shared understanding for dealing with early years obesity

Participants did not identify a common way of dealing with families where the diet was perceived to be unhealthy and infants and children were "at risk" or already overweight or obese. A range of different approaches were suggested which were not based on evidence or guidance.

Well postnatal, postnatal. So I think that would be a start, but then also through you've got mother and toddler groups, there's lots of groups that go on, parenting groups, and you know even at nurseries and things like that there's ways to get it in, education in. So I think education is really key. GP243.

*Something that helps them, so in other words it's supportive rather than sort of tell them off that they're doing something wrong, it is understand, just understand why it's happened and say you know what this is not entirely your fault and I would go through let me see how we can work together on this and see what we can do. GP252*.

There was a suggestion from practice nurses that any further expansion of their role around obesity prevention activities would require additional training.

*If it was sort of deemed that we should be doing it erm a bit more sort of training on how to have a structured approach. PN227*.

However, whilst participants tried to focus on appropriate early years interventions many of their responses were more suitable for childhood obesity management than primary prevention.

I think a programme of support, that's actually structured, and I know that that's what you're getting involved with, and I actually think that is what's most useful, and not just mostly focusing on activity really, rather than just on what they're eating because I think in an age where our kids generally sit and play computer games, the parents don't feel the kids are safe to be able to go out. GP239.

## Discussion

### Main findings

The survey revealed that GPs and nurses were less likely to be consulted at least weekly about infant feeding than nursery nurses and health visitors and were less confident about the advice they gave to parents despite being more knowledgeable about the health risks of obesity. Indeed HCPs' confidence in providing advice was unrelated to knowledge about obesity risk. GPs lower levels of confidence in providing infant feeding advice may be partially explained by the qualitative data in which they suggest that this is not their primary role and that training is not readily available. GPs and practice nurse believe that infant feeding advice is the health visitors' role. Consequently their advice giving around infant feeding is responsive and based on anecdotal or experiential knowledge. GPs report adopting a parent-centred approach and are wary of adversely affecting the doctor-parent relationship. This approach may constrain their attempts to identify infant obesity and/or improve parental feeding practices during routine consultations.

GPs and nurses report a low level of concern about infants who may be at risk of developing childhood obesity, despite their greater knowledge about the health risks associated with obesity. Infant overweight and obesity were not consistently recognised and a lack of common terminology combined with a dearth of training and guidance seemed to make identification and intervention difficult. The contrast between the greater knowledge of the health risks of obesity reported by the GPs and nurses, compared to nursery nurses and health visitors, might be explained by the qualitative data. GPs and practice nurses described the management of obesity rather than primary prevention and it seems likely that their greater knowledge will have emerged from the education and guidance they have received for their role managing adults who are overweight or obese. Health visitors and nursery nurses had lower levels of knowledge about the health risks of obesity, which may reflect an absence of exposure to training and guidance around obesity prevention and management.

### Strengths and limitations of study

Study strengths included the variety and number of HCPs who completed the ORK-10 scale. The ORK-10 has strong psychometric properties and has been successfully administered in similar populations, [27,29] though it primarily explores the impact of obesity on adult rather than child health. This study took place in two counties in the East Midlands, UK. There was a low response rate to the postal survey and therefore it was made available on-line with an invitation to complete it distributed by the PCRN and health visitor managers via email. The research team had no control over the distribution lists for these organisations; therefore a denominator for the number of HCPs who are likely to have received an email invitation to complete the survey on-line is not available. The response rate is likely to have been poor and given that both the survey and interview participants were volunteers the sample may have been biased towards HCPs with an interest in the subject. Therefore the findings may not be generalisable to HCPs working in other geographical locations in the UK. Thematic analysis is appropriate for the qualitative aspect of this study as it enables the researchers to identify, analyse and describe patterns in participants' experiences [34]. The interviews were undertaken by researchers who were also practising clinically as a health visitor and a paediatrician. Participants were aware of their clinical backgrounds and this may have influenced the interview content in that GPs and practice nurses may have felt that the interviewers were more knowledgeable than themselves about the subject. Data collection took place during 2008-2009 and there has subsequently been considerable media attention surrounding the timing of infant weaning; therefore HCP views may have altered since the study took place.

### Comparison with previous literature

No previous study has examined UK HCPs knowledge and GPs and practice nurses beliefs and current practice in relation to primary obesity prevention during infancy. GP and practice nurses' accounts that many infants are overfed are supported by literature pointing to widespread beliefs that much infant distress is triggered by hunger and that infants should finish a bottle of formula milk [26,37]. GPs and practice nurses' perceptions that health visitors should be delegated responsibility for infant feeding and weight management is endorsed by the findings of another study in which where parents believed that infant feeding advice is not the responsibility of practice staff [26]. Barriers to primary prevention were similar to those identified in studies exploring GPs' views about childhood obesity care [20,21]. These include GPs' conflicts around maintaining relationships with parents set against the need to raise the issue of obesity with overweight parents [38] and/or overweight children [20]. Paucity of training and guidance has previously been identified as a barrier to GPs' management of childhood obesity [20-22]. The lack of shared understanding about how to manage infants at risk and the communication barriers between HCPs particularly in relation to records about infants' diet, growth and weight is of concern as team working is crucial to successful prevention programmes [39].

### Implications for practice and future research

UK primary care tends to focus on the management of adult obesity although some areas of the country are beginning to systematically address the issue childhood obesity. US research suggests that infant overweight and obesity should be explicitly acknowledged as a health issue amongst HCPs [40] which would consequently allow guidelines, staff training and preventative initiatives. Given current system constraints and the absence of a coordinated multi-disciplinary approach, further research is needed to determine the best way of preventing and managing early years overweight and obesity consistently between and across health professional groups.

## Conclusions

There remain a number of challenges to enable a shift from a curative to a preventative focus for childhood obesity in primary care. Differences in competence, confidence and approach between HCPs who work with parents and young children are compounded by a limited shared 'language' and problems of interdisciplinary communication. Intervention is needed to improve health visitors and nursery nurses knowledge of obesity risk and HCPs capacity to identify and manage infants' at risk of developing childhood obesity. GPs value strategies that maintain relationships with vulnerable families and interventions to improve their advice-giving around infant feeding need to take account of this. Although health visitors may lead in this domain of care, a multidisciplinary team approach is needed as parents encounter a range of HCPs. Further research is needed to determine optimal ways of intervening with infants at risk of obesity in primary care.

## Competing interests

Sarah Redsell on behalf of all authors declares that SR, PA, DN, NS, JS, CG [and their spouses, partners, or children] have no relationships, financial, or non-financial interests with anyone that may be relevant to the submitted work.

## Authors' contributions

SR, DN, NS, JS, CG were involved in the study conception and design; PA, DN undertook the data collection; SR, PA, DN, NS, CG were involved in data analysis; SR, DN, NS, JS, CG contributed to the interpretation of the data. SR, PA, DN, NS, JS, CG contributed to drafting and revising the article. SR, PA, DN, NS, JS, CG have all approved the final version of the paper to be submitted for publication.

All authors had full access to the data.

## Pre-publication history

The pre-publication history for this paper can be accessed here:

http://www.biomedcentral.com/1471-2296/12/54/prepub
